# Post-carvedilol myocardial function in cats with obstructive hypertrophic cardiomyopathy

**DOI:** 10.3389/fvets.2025.1571850

**Published:** 2025-03-28

**Authors:** Takahiro Saito, Ryohei Suzuki, Yunosuke Yuchi, Haru Fukuoka, Shuji Satomi, Takahiro Teshima, Hirotaka Matsumoto

**Affiliations:** ^1^Laboratory of Veterinary Internal Medicine, School of Veterinary Medicine, Faculty of Veterinary Science, Nippon Veterinary and Life Science University, Tokyo, Japan; ^2^Sagamihara Animal Medical Center, Kanagawa, Japan; ^3^Garden Veterinary Hospital, Tokyo, Japan; ^4^Pet Clinic Lusty, Osaka, Japan

**Keywords:** beta-blocker, feline, left ventricular outflow tract obstruction, myocardium, two-dimensional speckle-tracking echocardiography, strain, systolic anterior motion of the mitral valve leaflet

## Abstract

**Introduction:**

Hypertrophic cardiomyopathy (HCM) is the most prevalent cardiac disease in cats, and one phenotype includes obstructive HCM with dynamic left ventricular outflow tract obstruction (DLVOTO). Myocardial function has been reported to be lower in cats with obstructive HCM than in non-obstructive HCM. Carvedilol, because of its pharmacological action, is expected to reduce the pressure gradient associated with DLVOTO, but no previous reports have studied its effects on myocardial function. This study aimed to evaluate myocardial function in cats with obstructive HCM with left ventricular outflow tract obstruction treated by carvedilol administration.

**Methods:**

This retrospective observational study included 16 cats with obstructive HCM and subsequent treatment of DLVOTO with carvedilol. In addition to conventional echocardiography, strain and strain rates in the left ventricular longitudinal and circumferential directions were measured using layer-specific two-dimensional speckle tracking echocardiography. Each variable was then compared before and after carvedilol medication.

**Results:**

Systolic anterior motion of the mitral valve disappeared in 14 cats and all cats showed resolved DLVOTO with maximal left ventricular outflow tract blood flow velocity of <2.5 m/s after carvedilol administration (*P* < 0.01). Circumferential strain in the epicardial layer and in the whole layer was significantly increased after carvedilol administration (*P* < 0.01, *P* = 0.04, respectively). In contrast, systolic longitudinal strain showed no significant difference between before and after carvedilol administration.

**Conclusion:**

Treatment of obstructive HCM with carvedilol improved DLVOTO and myocardial function without a negative inotropic effect. Carvedilol may be effective in treating cats with obstructive HCM.

## 1 Introduction

Hypertrophic cardiomyopathy (HCM), which is characterized by concentric hypertrophy of the left ventricular (LV) wall, is a highly prevalent heart disease in cats (1, 2). In addition, the detection of feline cardiomyopathy appears to have improved in recent years with the improved accuracy of diagnostic equipment, particularly in ultrasound imaging. The age at which HCM is diagnosed in cats varies, and its prognosis ranges from asymptomatic to congestive heart failure, arterial thromboembolism, and sudden death, even at a young age (3–7). HCM also shows diverse phenotypes, with some cases showing dynamic LV outflow tract obstruction (DLVOTO) associated with systolic anterior motion of the mitral valve (SAM). The presence of DLVOTO may cause increased afterload, exacerbation of LV wall thickening, abnormal relaxation, myocardial ischemia, and decreased single-beat output (8). In human patients with obstructive HCM, the presence of DLVOTO with LV outflow tract pressure gradient ≥50 mmHg is considered as a criterion for treatment because of its association with worse outcome (9). We have previously reported that LV myocardial function assessed using two-dimensional speckle-tracking echocardiography (2D-STE) is reduced in feline obstructive HCM with DLVOTO in comparison with that in non-obstructive HCM (10). Therefore, DLVOTO could be a therapeutic target also in feline HCM regardless of the presence of heart failure (2).

Carvedilol, a beta-blocker, blocks the action of catecholamines, resulting in improvement of diastolic dysfunction and suppression of myocardial ischemia associated with an increase in heart rate (11). Although the potential for myocardial function reduction due to the negative inotropic effect of carvedilol remains a concern, no previous report has examined the effect of carvedilol on myocardial function. We hypothesized that LV myocardial function would improve if DLVOTO were reduced by carvedilol administration in cats with obstructive HCM. Therefore, this study aimed to compare myocardial function indices before and after carvedilol treatment in cats with obstructive HCM with DLVOTO.

## 2 Methods

This retrospective observational study adhered to our university's animal care and use guidelines. The study was approved by our university's ethics committee (R2-4), and the owners gave adequate informed consent.

### 2.1 Animals

Medical records were reviewed from May 2015 to August 2022 to enroll cats diagnosed with obstructive HCM showing DLVOTO improvement with carvedilol administration. Each cat received a thorough clinical examination, blood pressure measurements, electrocardiographic evaluations, chest radiographs, and echocardiograms. The diagnostic criterion for HCM was a maximum LV wall thickness measuring ≥6 mm at end-diastole and distinct SAM were included in this study. A cat with SAM was defined as a case in which the anterior mitral leaflet of the mitral valve was in contact with the interventricular septum toward the end of systole in the right parasternal long-axis view. Obstructive HCM was defined as an LV outflow tract maximum blood flow velocity (LVOT *V*_max_) >2.5 m/s, based on previous reports (11, 12). In addition, patients were not administered beta-blockers at the time of diagnosis, which was a requirement for inclusion. Cats in this study started on carvedilol after diagnosis and had LVOT *V*_max_ < 2.5 m/s at the time of the return visit within 1 year. In cats that were already treated with medications other than carvedilol at diagnosis, no medication changes were made. Other types of cardiomyopathies were excluded by assessing the LV systolic function and wall thickness, which were found to be in the normal range using allometric scaling, according to previously published reports (2, 13). Cats with systemic diseases, hypertension, metabolic diseases, neoplastic diseases, or dehydration suggestive of secondary myocardial hypertrophy were excluded from the study. Cats with missing data were also excluded from this study.

### 2.2 Echocardiography

A single researcher (R.S.) performed conventional echocardiography using a Vivid E95 echocardiography scanner and 12S transducer (GE Healthcare, Tokyo, Japan). During the examination, a lead II electrocardiogram was obtained and continuously displayed on the screen. Non-sedated cats were manually restrained in the left and right lateral recumbency. The data were acquired for a minimum of five heartbeats. Subsequently, echocardiographic data analysis was performed by a single researcher (T.S.) using an offline workstation (EchoPAC PC, version 204, GE Healthcare, Tokyo, Japan) on a different day from image acquisition.

Various measurements were taken from the echocardiographic images. To ensure accuracy, each measurement was averaged over three consecutive cardiac cycles before conducting the analysis. The left atrial-to-aortic diameter ratio was determined from the right parasternal short-axis view at the basal heart level. Additionally, end-diastolic interventricular septal thickness (IVSd), LV end-diastolic posterior wall thickness (LVPWd), LV end-diastolic internal diameter (LVIDd), LV end-systolic internal diameter, and fractional shortening (FS) were measured from the right parasternal short-axis view at the level of the chordae tendineae. Relative LV wall thickness (RWT) was calculated using the following formula (14, 15):


$$RWT=\frac{\left(IVSd+LVPWd\right)}{LVIDd}$$

Pulsed-wave Doppler was used to measure the transmitral inflow in the left apical four-chamber view, where the peak velocities of the early diastolic wave (E-wave) and late diastolic wave (A-wave) were determined. The E-wave to A-wave velocity ratio (E/A) was calculated.

The pulsed-tissue Doppler technique was used to obtain the velocity of the mitral annular motion in the left apical four-chamber view, specifically from the interventricular septum. Three parameters were measured: Peak velocity of systolic mitral annular motion (s′), peak velocity of early diastolic mitral annular motion (e′), and peak velocity of late diastolic mitral annular motion (a′).

As described previously (12, 16–19), 2D-STE analysis of cats is performed with high-quality images obtained using conventional echocardiography. To evaluate circumferential deformations using 2D-STE, a right parasternal short-axis view of the LV at the level of the chordae tendineae level was used (12, 20). A left apical 4-chamber view was used to analyze the longitudinal deformations. Peak systolic strain was measured in the longitudinal and circumferential directions (SL and SC, respectively) (21). The SL and SC were measured individually for the endocardial, epicardial, and whole layer. Additionally, early-diastolic and late-diastolic strain rate in the longitudinal and circumferential direction were measured (SrL E, SrL A, SrC E, and SrC A, respectively). The early to late-diastolic strain rate ratio (E/A) was calculated.

Our laboratory has previously reported observer variability in 2D-STE (12, 18, 19, 21).

### 2.3 Statistical analysis

All variables are presented as medians (interquartile ranges). For the statistical analysis, we used commercially available software (R 2.8.1; https://www.r-project.org/). Data were assessed for normality using the Shapiro–Wilk test and compared before and after carvedilol administration. Normally distributed data were evaluated with a paired *t*-test, and non-normally distributed data were evaluated using a Wilcoxon rank-sum test. *P*-values < 0.05 were considered significantly different.

## 3 Results

### 3.1 Clinical profiles

The demographic characteristics, duration of carvedilol administration and dosage, and physical examination findings before and after carvedilol administration are summarized in Table 1. All cats were given carvedilol twice daily. The duration of carvedilol administration was the same as the duration of the before and after comparisons. Sixteen cats were included in the study, and no cases of severe congestive heart failure, such as pulmonary edema or pleural effusion, were observed. Medications other than carvedilol were angiotensin-converting enzyme inhibitors (*n* = 1), calcium channel blockers (*n* = 1), pimobendan (*n* = 1), and antithrombotic agents (*n* = 3). The heart rate showed a significant decrease before and after carvedilol administration. The systolic blood pressure showed no significant difference after the administration of carvedilol.

**Table 1:** Clinical characteristics in cats with obstructive hypertrophic cardiomyopathy before and after carvedilol administration.

**Variables**			***P*-value**
N (male/female)	16 (9/7)		
Age (years)	1.9 (1.0, 3.3)		
ACVIM (B1, B2, C/D)	11, 5, 0		
**Carvedilol**			
Period of administration (day)	48 (26, 92)		
Dose (mg/kg/day)	0.2 (0.1, 0.5)		
Body weight (kg)	4.0 (3.3, 5.1)	4.2 (3.7, 5.2)	0.90
Heart rate (bpm)	194 (177, 219)	172 (158, 183)^*^	< 0.05
Systolic blood pressure (mmHg)	126 (120, 149)	142 (130, 144)	0.47

Data are presented as median (interquartile range).

^*^Significantly different values compared to before carvedilol administration.

### 3.2 Echocardiography

**Figure 1:**
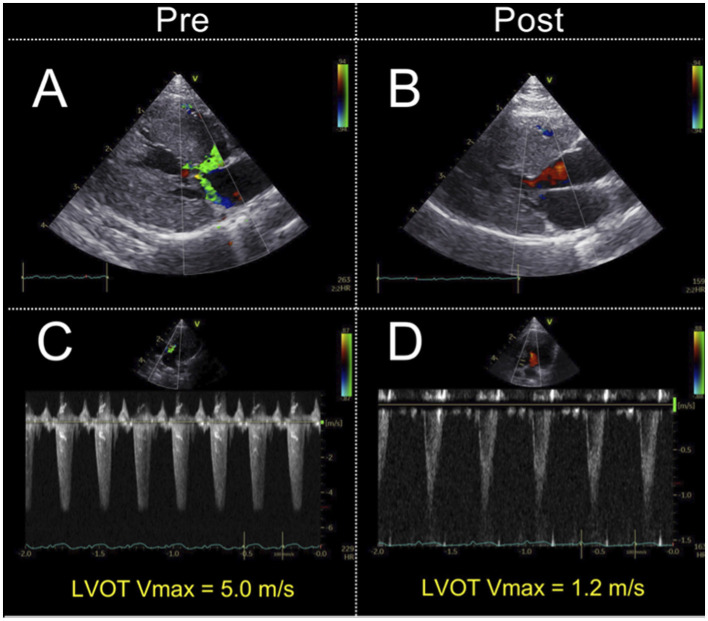
Echocardiographic findings in a representative case of systolic anterior motion of the mitral valve resolved by carvedilol administration. Echocardiographic findings on color Doppler. The images show the absence of mosaic on the left ventricular outflow tract and mitral regurgitation **(A, B)**. Doppler echocardiography shows the maximal left ventricular outflow tract blood flow velocity decreased **(C, D)**. LVOT V_max_, left ventricular outflow tract maximum blood flow velocity.

The conventional echocardiographic variables are summarized in Table 2. The IVSd, LVPWd, LV maximum wall thickness, and *a*' significantly decreased after carvedilol administration. The LVIDd showed no significant difference after administration of carvedilol. Although SAM was present in all cats at the initial evaluation, it disappeared in 14 cats after carvedilol administration. Two cats showing fusion of E- and A-waves were excluded from the analysis. Echocardiographic findings in a representative case of SAM resolved by carvedilol administration are shown in Figure 1.

**Table 2:** Results of conventional echocardiographic variables in cats with obstructive hypertrophic cardiomyopathy before and after carvedilol administration.

**Variables**	**Pre**	**Post**	***P*-value**
LA/Ao	1.2 (1.1, 1.6)	1.2 (1.1, 1.3)	0.12
IVSd (mm)	6.2 (5.3, 7.2)	5.8 (4.1, 6.6)^*^	< 0.05
LVPWd (mm)	6.0 (4.8, 6.5)	5.2 (4.5, 6.1)^*^	< 0.05
LVIDd (mm)	12.8 (12.1, 14.2)	14.0 (12.0, 14.8)	0.31
LV maximum wall thickness (mm)	6.8 (6.0, 7.5)	6.4 (5.7, 7.0)^*^	< 0.05
RWT	0.9 (0.8, 1.1)	0.8 (0.7, 0.9)^*^	0.08
FS (%)	51.4 (45.4, 58.8)	50.0 (43.8, 56.1)	0.65
*s*′ (cm/s)	8.1 (6.2, 10.3)	6.8 (5.7, 7.5)	0.07
*e*′ (cm/s)	4.6 (4.2, 5.3)	4.8 (3.4, 5.7)	0.12
*a*′ (cm/s)	9.2 (7.0, 10.6)	7.0 (6.4, 7.8)^*^	< 0.01
LVOT V_max_	3.9 (3.6, 4.8)	1.2 (1.0, 1.3)^*^	< 0.01

LA/Ao, left atrial-to-aortic diameter ratio; IVSd, end-diastolic interventricular septal thickness; LV, Left ventricular; LVPWd, end-diastolic LV free-wall thickness; LVIDd, end-diastolic LV internal diameter; RWT, relative left ventricular wall thickness; FS, fractional shortening; s′, peak velocity of systolic mitral annular motion; e′, peak velocity of early diastolic mitral annular motion; a′, peak velocity of late diastolic mitral annular motion; LVOT V_max_, peak velocity of left-ventricular outflow-tract.

Data are presented as median (interquartile range).

^*^Significantly different values compared to before carvedilol administration.

**Figure 2:**
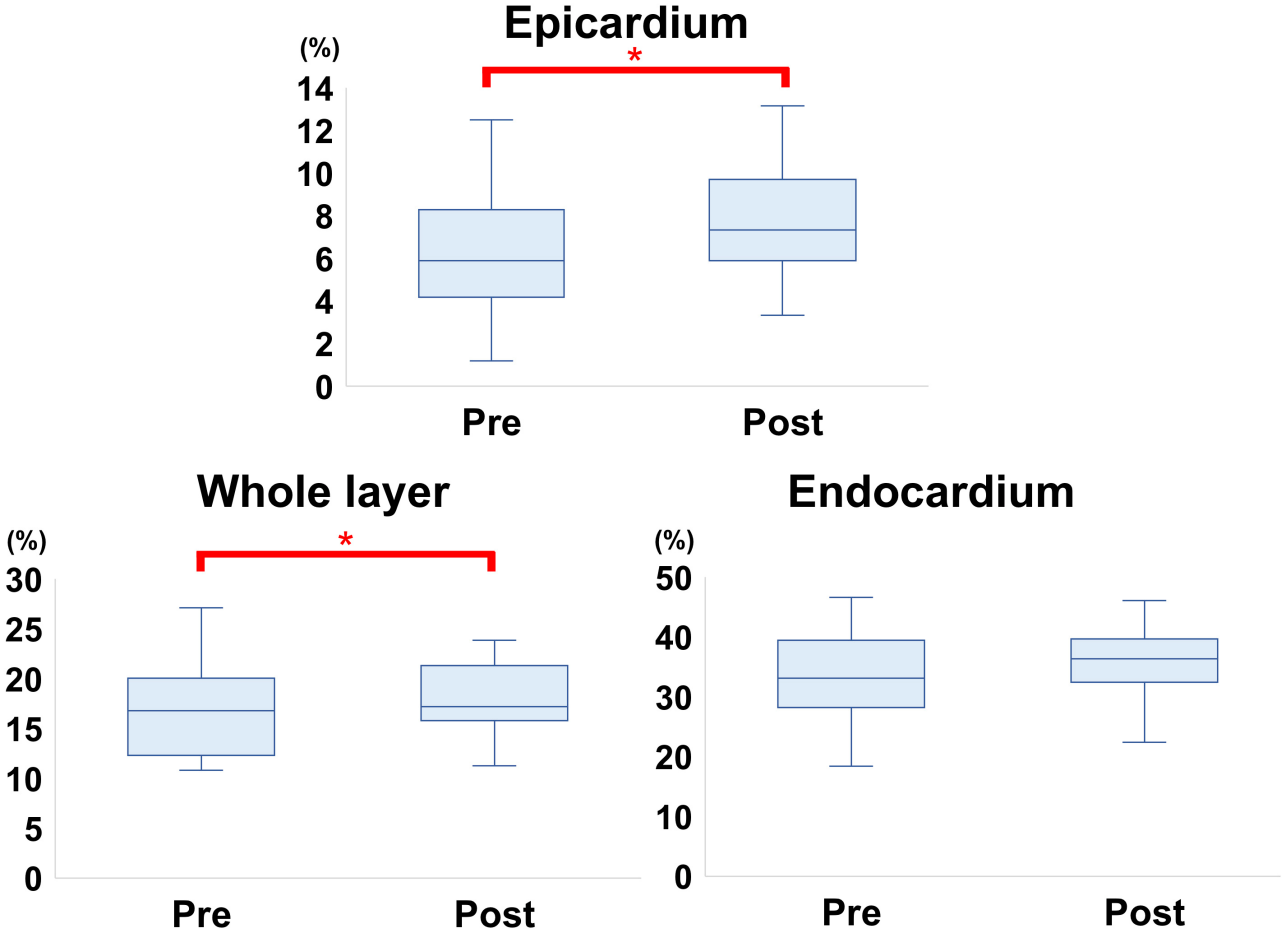
Comparison of circumferential strain evaluated by two-dimensional speckle-tracking echocardiography before and after carvedilol administration (box-and-whisker diagram). In the epicardium and whole layer, the circumferential strain shows significant differences before and after carvedilol administration. *Significantly different values compared to before carvedilol administration.

**Figure 3:**
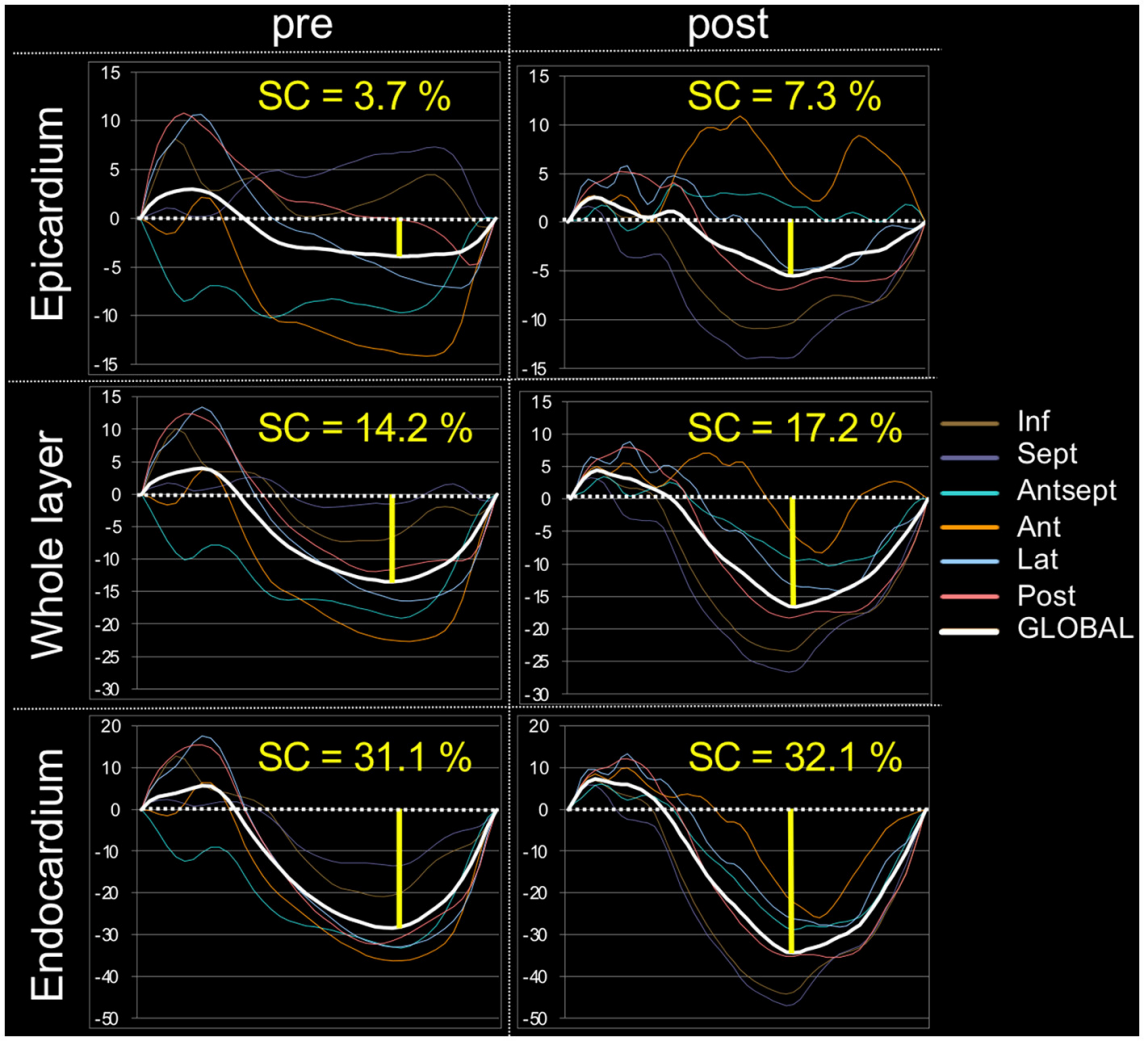
Myocardial circumferential analysis evaluated using two-dimensional speckle-tracking echocardiography in a representative case of systolic anterior motion of the mitral valve resolved by carvedilol administration. These are representative data, showing a stronger increase in circumferential strain after carvedilol administration than before in all layers in this case. SC, circumferential strain.

The myocardial strain results of the analysis using 2D-STE are summarized in Table 3. The SL did not show significant differences before and after carvedilol administration in any of the layers. In the SC, the endocardial layer showed no significant difference after administration of carvedilol. In contrast, the SC in the epicardial layer and in the whole layer increased significantly after carvedilol administration (Figure 2). Myocardial circumferential analysis evaluated by 2D-STE in a representative case of SAM resolved by carvedilol administration is shown in Figure 3. Diastolic myocardial strain rates assessed by 2D-STE are summarized in Table 4. The SrL A decreased significantly after carvedilol administration. On the other hand, there were no significant differences in indices other than SrL before and after carvedilol administration.

**Table 3:** The myocardial strain assessed using two-dimensional speckle tracking echocardiography in cats with obstructive hypertrophic cardiomyopathy before and after carvedilol administration.

**Variables**	**Pre**	**Post**	***P*-value**
**SL (%)**			
Whole layer	19.0 (12.3, 20.4)	15.3 (14.0, 20.8)	0.82
Endocardium	23.6 (19.1, 25.5)	19.6 (18.3, 25.9)	0.82
Epicardium	14.6 (9.7, 17.7)	12.8 (10.3, 17.1)	0.85
**SC (%)**			
Whole layer	16.9 (12.3, 21.1)	17.2 (15.8, 19.3)^*^	0.03
Endocardium	34.0 (28.9, 40.2)	35.9 (32.3, 40.1)	0.19
Epicardium	6.2 (4.1, 8.4)	7.2 (5.7, 7.8)^*^	< 0.01

SL, peak systolic strain in the longitudinal direction; SC, peak systolic strain in the circumferential direction.

Data are presented as median (interquartile range).

^*^Significantly different values compared to before carvedilol administration.

**Table 4:** The myocardial strain rate assessed using two-dimensional speckle tracking echocardiography in cats with obstructive hypertrophic cardiomyopathy before and after carvedilol administration.

**Variables**	**Pre**	**Post**	**P-value**
SrL E	1.7 (1.0, 3.3)	2.6 (1.5, 3.9)	0.40
SrL A	3.3 (2.6, 5.0)	2.2 (1.7, 2.6)^*^	0.03
SrL E/A	0.8 (0.2, 1.2)	1.4 (0.8, 2.0)	0.11
SrC E	2.3 (1.2, 2.6)	2.5 (1.6, 3.3)	0.78
SrC A	2.6 (2.0, 3.2)	2.0 (1.6, 2.4)	0.31
SrC E/A	0.8 (0.3, 1.8)	1.3 (0.8, 2.0)	0.58

SrL E, early-diastolic strain rate in the longitudinal direction; SrL A, late-diastolic strain rate in the longitudinal direction; SrC E, early-diastolic strain rate in the circumferential direction; SrC A, late-diastolic strain rate in the circumferential direction.

Data is presented as median (interquartile range).

^*^Significantly different values compared to before carvedilol administration.

## 4 Discussion

This is one of the few reports highlighting the effects of carvedilol administration on myocardial function evaluated by 2D-STE in cats with obstructive HCM. Cats with obstructive HCM were found to have improved myocardial function after treatment with carvedilol, without worsening the condition due to negative inotropic effect, which was an initial concern. The results of this study suggest that carvedilol may be useful as a treatment for cats with obstructive HCM.

This study focused on myocardial function in cats with obstructive HCM whose DLVOTO improved with carvedilol. In veterinary medicine, atenolol is the most discussed beta-blocker for treating feline obstructive HCM. Atenolol selectively acts on β_1_ receptors, which inhibits vasodilating effect through β_2_ receptors blockade. However, a recent study has reported that atenolol could not improve 5-year survival in cats (22), although atenolol could improve DLVOTO (11). One concern using atenolol is that this drug is water-soluble, which results in slower gastrointestinal absorption and less transfer to the central nervous system compared to fat-soluble drugs. Therefore, fat-soluble beta-blocker (e.g., carvedilol and bisoprolol) is used preferentially in human obstructive HCM. Carvedilol is one of the fat-soluble, non-selective beta-blockers. As a difference from other beta-blockers, carvedilol has been reported to reduce the risk of developing fatal arrhythmias (23). Additionally, carvedilol has antioxidant and apoptosis-inhibiting effects (24). Although this study could not evaluate these effects in detail, they might contribute to the improvement of myocardial function in cats with obstructive HCM. However, carvedilol also has α_1_ receptors blockade as well as the non-selective β receptors blockade. Hence hypotension due to vasodilating effect is a main concern as a side effect. However, systemic blood pressure of this study showed no significant change after carvedilol administration. The main factor attributed to the results was possibly due to increased cardiac output through the alleviation of DLVOTO. Therefore, our results indicate that carvedilol might be a safe treatment option for feline obstructive HCM.

Heart rate of this study was significantly decreased with carvedilol administration. This may be due to the negative chronotropic effects of carvedilol (25, 26). The IVSd, LVPWd, and LV maximum wall thickness were also significantly lower following carvedilol administration. This may be a result of the prolonged LV relaxation time that occurs as the heart rate decreases, which promotes the elongation of the LV wall. Previous reports have shown a significant increase in LVIDd and FS following carvedilol administration in cats with HCM, owing to the negative chronotropic effect of carvedilol (17). In this study, SAM disappeared in many cases, the distinct DLVOTO disappeared in all cases, and the LVOT *V*_max_ was significantly lower after carvedilol administration. In previous reports, SAM has been reported to be caused by thickening of the LV wall and elongation of the mitral valve leaflet, resulting in a decrease in the relative distance between the papillary muscle and the mitral valve leaflet (27). In addition, early systolic contact of the mitral valve with the ventricular septum increases the pressure gradient. This gradient is thought to press the mitral valve against the ventricular septum, further narrowing the left ventricular outflow tract and thus increasing the pressure gradient (28). Although this study did not include a morphological evaluation of the mitral valve, the relatively thinner LV wall observed with increased LV volume at end-diastole and decreased heart rate may have reduced the incidence of SAM and improved DLVOTO.

We have previously reported a lower systolic SC in cats with obstructive HCM and DLVOTO (10), and the results of this study were consistent with this observation. This lower SC may have been caused by the increased pressure gradient and decreased cardiac output associated with DLVOTO. In addition, previous studies on HCM in humans reported that survival was shorter in HCM with DLVOTO than in HCM without DLVOTO (29). This may be because an increase in LV pressure owing to obstruction causes increased wall stress, myocardial ischemia, and fibrosis, resulting in diastolic dysfunction and sudden death, thus DLVOTO may worsen cardiac function (29). Furthermore, previous studies have reported that decreased SC in the epicardium of cats with HCM reflects decreased myocardial function due to layer-to-layer compensatory interactions (19, 30). Therefore, the present study can be interpreted as a significant increase in myocardial function, especially in SC on the epicardium after carvedilol administration, resulting in an increase in SC in the whole layer accompanied with an improvement in systolic myocardial function.

In cats with HCM, SL is known to be reduced early and is even lower in cats with more severe HCM (18, 31). In addition to histopathological changes, such as altered myocardial fiber orientation, compensatory mechanisms in the myocardium are thought to be associated with myocardial dysfunction (12, 18, 32). In human patients with HCM, decreased SL in the whole layer has been reported to be a poor prognostic factor, even when FS is normal (33). Although the SL before carvedilol administration in this study was considered to be lower than that in healthy cats, no significant difference was observed in the SL before and after carvedilol administration. The results of SL indicate that carvedilol did not contribute to the worsening of the disease state.

Unlike the results of SL, SC increased significantly after carvedilol administration. The improvement in DLVOTO after carvedilol administration caused by prolonging the diastolic filling time and LV ejection acceleration time might have improved myocardial function, especially in the circumferential direction, by reducing LV pressure overload and cardiac output failure. The discrepancy in results for SL and SC might be due to compensatory mechanisms of myocardial motion. A previous study reported that circumferential function compensates for impaired longitudinal myocardial function in human patients with cardiovascular risk factors (34). Our results indicate that DLVOTO might be associated with compensatory myocardial motion, particularly in the circumferential direction, also in cats with obstructive HCM. The unchanged loading indices such as LVIDd, LA/Ao, and systemic blood pressure before and after carvedilol administration emphasize that the improvement in SC could reflect the improvement in precise myocardial function. In addition to the alleviation of DLVOTO, the following changes in loading conditions associated with carvedilol administration might have also contributed to the improvement in SC. First, the specific α_1_ receptors blockade of carvedilol might have contributed to further improvements in SC through the reduction of peripheral vascular resistance. Moreover, the decreased heart rate and prolonged diastole may have also promoted reduced myocardial oxygen consumption and improved coronary blood flow through the sinus of Valsalva. Studies on beta-blocker (metoprolol) treatment of HCM with DLVOTO in humans have also reported that beta-blockers improve myocardial strain by decreasing DLVOTO at rest and during exercise, prolonging diastolic filling time and increasing myocardial fiber elasticity (35). Overall, carvedilol could theoretically reduce cardiac function due to its negative inotropic effect, but according to the results of this study and previous reports, it may be effective in cats with obstructive HCM with improved DLVOTO.

Among diastolic indices assessed by the 2D-STE, SrL A significantly increased after carvedilol administration. A previous study reported that speckle tracking-derived SrL E and SrL A are significantly decreased in cats with HCM compared to healthy controls (36). Although there was no significant increase in SrL E, the results of this study may suggest that carvedilol administration may have improved diastolic myocardial function, which is already reduced in cats with HCM. In addition, left atrial function is closely linked to LV diastolic function, and enhancement of left atrial function is thought to be a compensatory mechanism for the progression of LV diastolic dysfunction (37, 38). If left atrial pressure is in the normal range, atrial function is considered enhanced to maintain LV filling pressure. Therefore, it is possible that the significant SrL A reduction in this study may have eliminated the compensation for diastolic dysfunction.

In previous studies, tissue Doppler-derived *s*' and *e*' were significantly lower in the HCM cat groups treated with beta-blockers (atenolol) than in those treated with placebo (39). However, in the present study, there was no significant decrease in *s*' and *a*' after carvedilol administration. In addition, the 2D-STE in this study showed no declines in both the longitudinal systolic and diastolic function indices. Although the previous study did not note about DLVOTO, the improvement in DLVOTO by carvedilol administration observed in this study might have prevented the worsening of the tissue Doppler-derived indices through the alleviation of pressure overload. Furthermore, Atenolol is classified as a non-vasodilating beta-blocker and primarily reduces ventricular systolic function and cardiac output. Carvedilol, on the other hand, is classified as a vasodilating beta-blocker that decreases peripheral vascular resistance and increases cardiac output (40). These differences in mechanism of action may have led to the difference in results between atenolol and carvedilol. In addition, the method of 2D-STE overcomes the angle-dependent weakness of tissue Doppler and assesses not only local myocardial function, but also wall motion coordination, dyssynchrony, ischemia, and whole myocardial function (41). The improvement in SC in this study may reflect improved myocardial function for the reasons discussed above.

This study has several limitations. First, this study is a retrospective analysis of clinical cases. Furthermore, this study only included cats with obstructive HCM that had responded to carvedilol treatment. There are a certain number of cats that do not respond to carvedilol treatment. Therefore, the present results may not be applicable in such cases. Further studies are expected to evaluate the response rate of carvedilol to DLVOTO and the resulting improvement in clinical symptoms and survival period in a larger population. Additionally, this study included only cats with asymptomatic obstructive HCM (ACVIM stage B). Further studies are needed to evaluate the efficacy of carvedilol on cats with more progressed HCM. Second, the duration of carvedilol dosing and dosage in the study animals were not consistent and may have influenced the results of the study. In addition, medications already administered at the time of diagnosis could have affected the results. Third, this study did not perform a priori power calculation. A relatively small sample size of this study might affect statistical power. However, this study reported detailed myocardial function analysis in each case using the 2D-STE method. Fourth, this study did not perform the adjustment of statistical analysis. Therefore, there might be some type I error. Finally, this study was based on clinical rather than pathological diagnoses. In some cases, the diagnosis of cardiomyopathy may be incorrect or does not reflect the degree of myocardial damage.

In conclusion, DLVOTO in cats with obstructive HCM may worsen myocardial function assessed by layer-specific 2D-STE analysis. Carvedilol treatment may improve myocardial function, especially in the circumferential direction, without decreasing it. Although larger studies are needed, carvedilol may be a useful drug for improving myocardial function in cats with obstructive HCM.

## Data Availability

The original contributions presented in the study are included in the article/supplementary material, further inquiries can be directed to the corresponding author.
